# A population-based study of the extent of colorectal cancer screening in men with HIV

**DOI:** 10.1186/s12913-015-0711-9

**Published:** 2015-02-01

**Authors:** Tony Antoniou, Nathaniel Jembere, Refik Saskin, Alexander Kopp, Richard H Glazier

**Affiliations:** Department of Family and Community Medicine, St. Michael’s Hospital and University of Toronto, Toronto, ON Canada; St. Michael’s Hospital, The Li Ka Shing Knowledge Institute, Toronto, ON Canada; Institute for Clinical Evaluative Sciences, Toronto, ON Canada; Institute of Health Policy, Management and Evaluation, University of Toronto, Toronto, ON Canada; Centre for Research on Inner City Health, St. Michael’s Hospital, Toronto, ON Canada

**Keywords:** HIV, Colonoscopy/utilization, Colorectal neoplasms/diagnosis, Population surveillance, Health services accessibility, Male

## Abstract

**Background:**

Because of the increased life-expectancy of persons with HIV, the need for age-appropriate colorectal cancer screening among these patients will increase. We examined rates of colorectal cancer screening among HIV-infected men aged 50 to 65 years.

**Methods:**

We used Ontario’s administrative databases to identify all men between the ages of 50 and 65 years who were alive on April 1, 2007, and identified HIV-infected men using a validated case-finding algorithm. We excluded men with a history of colorectal cancer, anal cancer, inflammatory bowel disease and any colorectal investigation in the preceding five-years, and used multivariable regression to compare rates of colorectal cancer screening between men with and without HIV during five years of follow-up.

**Results:**

We identified 743,801 men between the ages of 50 and 65 years, of whom 1,432 (0.19%) were HIV-infected. The proportions of men with and without HIV who underwent any screening during the 5-year follow up period were 49.1% (95% CI 46.5% to 51.7%) and 41.4% (95% CI 41.3% to 41.5%), respectively. Compared with HIV-negative men, men with HIV had lower rates of fecal occult blood testing [adjusted rate ratio (aRR) 0.74; 95% confidence interval (CI) 0.63 to 0.87] and barium-enema radiography (aRR 0.66; 95% CI 0.39 to 1.12), but higher rates of colonoscopy (aRR 1.24; 95% CI 1.13 to 1.37), flexible sigmoidoscopy (aRR 1.72; 95% CI 1.28 to 2.30) and rigid sigmoidoscopy (aRR 2.98; 95% CI 2.26 to 3.93).

**Conclusion:**

As with the general population of men aged 50 to 65 years, less than half of the population of men with HIV received colorectal cancer screening. Strategies are required to improve uptake of this intervention.

**Electronic supplementary material:**

The online version of this article (doi:10.1186/s12913-015-0711-9) contains supplementary material, which is available to authorized users.

## Background

Rates of acquired immunodeficiency syndrome (AIDS)-associated malignancies such as Kaposi’s sarcoma and non-Hodgkin’s lymphoma have declined markedly among persons with human immunodeficiency virus (HIV) infection in the years following the introduction of combination antiretroviral therapy [1,2]. However, the incidence of cancers not traditionally associated with HIV, including colorectal cancer, has increased more than three-fold in this population over the same period, such that these non-AIDS-defining malignancies now account for an increasing proportion of deaths among persons with HIV [2-4]. In this context, research examining access to and utilization of services directed towards the prevention and treatment of cancer is required to optimize the health of these patients.

Colorectal cancer is the second leading cause of cancer-related death in North America, with an estimated 9,200 Canadians dying from this disease in 2012 [5]. While the natural history and prognosis of colorectal cancer in the setting of HIV remains poorly characterized, available data suggest that patients with HIV have more advanced stage disease at presentation relative to non-HIV infected individuals [6-10]. In light of these data, an aging cohort of persons with HIV and evidence that screening for colorectal cancer reduces mortality associated with this disease, integration of age-appropriate colorectal cancer screening into the care of patients with HIV will be increasingly required [11-13]. Although several studies suggest that colorectal cancer screening is underutilized in HIV-infected relative to non-infected patients, there are no population-based data examining the utilization of colorectal cancer screening in persons with HIV [14-18]. We therefore compared rates of colorectal cancer screening among 50 to 65 year old men living with and without HIV infection in Ontario, Canada, a setting of universal health coverage and home to over 40% of Canada’s population of persons with HIV [19]. We focused our comparisons on men because women comprise less than 15% of persons with HIV over the age of 50 in Ontario [20].

## Methods

### Data sources

We obtained data from Ontario’s administrative healthcare databases, which are available at the Institute for Clinical Evaluative Sciences through a data sharing agreement with the Ontario Ministry of Health and Long-Term Care. Specifically, we used the Ontario Health Insurance Plan database to identify claims submitted by physicians to the provincial universal health insurance program. We obtained diagnostic and procedural information on all patients discharged from hospitals and same-day surgery units from the Canadian Institute for Health Information Discharge Abstract Database. We used the Ontario Cancer Registry, a registry of all Ontario residents who have been diagnosed with or died of cancer, to identify individuals with a diagnosis of colorectal cancer. Finally, we used the Registered Persons Database, a registry of all Ontario residents eligible for provincial health services, to identify individual demographic information such as age and postal code, and the Institute for Clinical Evaluative Sciences Physician Database to determine physician specialty. These databases were deterministically linked in an anonymous fashion using encrypted health card numbers, and are routinely used for population-based research examining the utilization of colorectal investigations [21-23].

### Study population

We used the Registered Persons Database to identify all men in Ontario between the ages of 50 to 65 years who were alive and eligible for health insurance as of the index date of the study, April 1, 2007. From within this cohort, we identified men with HIV using a previously validated case-finding algorithm, the development of which has been described in detail elsewhere [24]. Briefly, an algorithm of three physician claims with an International Classifications of Diseases, 9^th^ edition (ICD-9) code for HIV infection (042, 043, 044) within a three year period achieved a sensitivity and specificity of 96.2% (95% confidence intervals: 95.2% to 97.9%) and 99.6% (95% confidence interavals: 99.1% to 99.8%), respectively, for the identification of persons with diagnosed HIV. Because our administrative databases do not allow us to distinguish between diagnostic and screening investigations, we excluded all men where the likelihood of receiving a colorectal cancer investigation for diagnostic reasons was high using an approach similar to that described in previous studies [22,25]. Specifically, we excluded men with a diagnosis of colorectal cancer, anal cancer and inflammatory bowel disease in the five years preceding the index date, and men who had received any colorectal investigation (i.e. fecal occult blood test, barium enema radiography, rigid or flexible sigmoidoscopy and colonoscopy) in the five years preceding the index date (see Additional file 1: Table S1 and S2 for relevant diagnostic and procedure codes). The remaining individuals approximated a cohort at average risk of colorectal cancer [25].

### Outcomes

The primary outcome of the study was the receipt of individual colorectal cancer screening during the five-year follow-up period. In Ontario, biennial fecal occult blood testing is the recommended screening modality for average risk individuals aged 50 to 74 years, with endoscopic investigations recommended for higher risk individuals or as follow-up to positive fecal occult blood tests. All colorectal screening tests (fecal occult blood testing and endoscopic tests) are provided at no cost to Ontario residents through the single-payer, government administered public health system. In this study, we examined rates of fecal occult blood testing and endoscopic investigations in the event that restricting our focus on the former would underestimate the extent of colorectal screening. We considered an individual appropriately screened if they received a fecal occult blood test within 2 years of cohort entry, or any one of a colonoscopy, flexible or rigid sigmoidoscopy, or barium-enema radiography examination within 5 years of cohort entry (see Additional file 1 for procedural codes). We followed each person in the cohort for up to five-years from the index date until the receipt of a colorectal investigation, death, or end of the study period (March 31, 2012), whichever occurred first. Consequently, each individual could only receive one colorectal investigation during the follow-up period.

### Statistical analysis

In our main analysis, we used multivariable Poisson regression analysis to investigate rates of colorectal cancer screening in men with and without HIV-infection. Adjusted rate-ratios comparing screening among men with and without HIV-infection were derived from models that included the natural logarithm of the count of each colorectal investigation as the dependent variable, the natural logarithm of person-time as an offset, and independent variables that may influence the receipt of colorectal cancer screening, including age, neighborhood income quintile based on recent postal code and 2006 Census data, urban versus rural residence, number of physician visits in the 2 years preceding cohort entry, and whether the individual had been seen by a gastroenterologist in the 2 years preceding cohort entry. We also used the Johns Hopkins Adjusted Clinical Groups Case-Mix System to adjust for differences in comorbidity burden and resource use in the two years preceding the index date [26]. This system uses diagnostic information from administrative databases to describe and predict use of health care resources. In this study, we used Aggregated Diagnosis Groups (ADGs), which are clusters of diagnostic codes that are similar in terms of severity and expected persistence. The number of ADGs ranges from 0 to a maximum of 32, with a higher number reflecting a higher level of co-morbidity. We also used Resource Utilization Bands (RUBs), which are aggregations of age-sex diagnostic groups associated with different levels of expected resource use, ranging from 0 (lowest expected health care use) to 5 (highest expected health care use), to categorize patients based on their expected use of health care resources. This system has been validated for use in Canadian populations, and both measures are routinely used for case-mix adjustment in health services research [27-29]. In a sensitivity analysis, we replicated these analyses in a cohort that included men who had undergone a colorectal cancer investigation in the five years preceding the index date, as these individuals may exhibit different health seeking behaviours than men who did not receive an investigation during this period.

In secondary analyses, we determined predictors for the receipt of colonoscopy and fecal occult blood testing in men with HIV only. The variables we included in these models were age, income quintile, rural or urban residence, level of co-morbidity reflected by the number of ADGs and RUBs, number of physician visits in the 2 years preceding cohort entry, and whether the individual had been seen by a gastroenterologist in the 2 years preceding cohort entry

All statistical analyses were conducted using SAS version 9.3 (SAS institute, Cary, North Carolina, USA).

### Ethics approval

We obtained ethics approval for this study from the Research Ethics Board of Sunnybrook Health Sciences Centre.

## Results

We identified a total of 725,801 men between the ages of 50 and 65 years who were alive as of April 1, 2007, of whom 1,432 (0.19%) were HIV-positive. Compared with men without HIV, men living with HIV were disproportionately represented in low income neighborhoods, had a greater comorbidity burden as reflected by the number of ADGs and RUBs, and had more physician visits in the two years preceding the index date (Table 1).

**Table 1:** **Baseline characteristics**

**Variable**	**HIV(n = 1,432)**	**No HIV(n =742,369)**	**p-value**
Mean age (SD)	54.9 (4.3)	56.2 (4.5)	<0.001
Rural residence, No. (%)	71 (5.0%)	110,943 (14.9%)	<0.001
Gastroenterologist visit in previous 2 years, No. (%)	82 (5.7%)	14,644 (2.0%)	<0.001
Mean number of physician visits in previous 2 years (SD)	21.9 (21.3)	9.1 (11.9)	<0.001
Number of Aggregated Diagnosis Groups, No. (%)			<0.001
0	38 (2.7%)	116,677 (15.7%)	
1 to 3	370 (25.8%)	271,294 (36.5%)	
4 to 7	624 (43.6%)	265,225 (35.7%)	
8 to 10	249 (17.4%)	66,737 (9.0%)	
>11	151 (10.5%)	22,436 (3.0%)	
Number of Resource Utilization Bands, No. (%)			<0.001
0	38 (2.7%)	116,685 (15.7%)	
1	7 (0.5%)	34,908 (4.7%)	
2	20 (1.4%)	128,212 (17.3%)	
3	698 (48.7%)	366,400 (49.4%)	
4	403 (28.1%)	66,170 (8.9%)	
5	266 (18.6%)	29,994 (4.0%)	
Income Quintile, No. (%)^†^			<0.001
1 (lowest)	452 (31.6%)	144,953 (19.5%)	
2	304 (21.2%)	148,317 (20.0%)	
3	229 (16.0%)	145,079 (19.5%)	
4	189 (13.2%)	147,895 (19.9%)	
5	233 (16.3%)	148,757 (20.0%)	
Missing	25 (1.7%)	7,368 (1.0%)	

^**†**^Mean household income of neighborhood.

Aggregated Diagnosis Groups: Measures levels of patient co-morbidity, with increasing number representing higher level of comorbidity burden.

Resource Utilization Bands: Measures category of expected resource use, from 0 (lowest expected resource use) to 5 (highest expected resource use).

**Figure 1:**
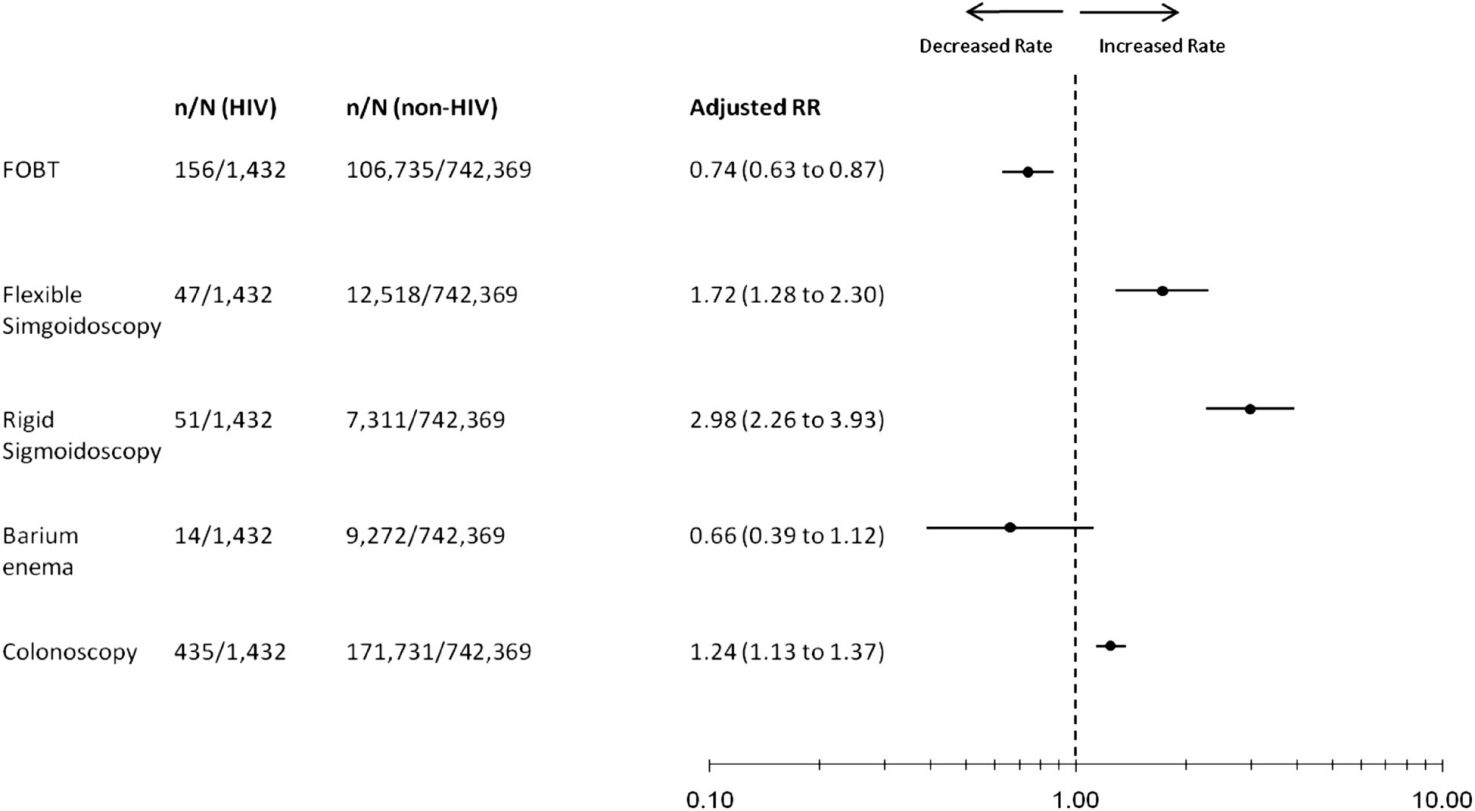
**Adjusted rate ratios for receipt of colorectal investigations in men with HIV relative to non-HIV-infected men.**

Overall, 703 (49.1%; 95% confidence interval 46.5% to 51.7%) HIV-infected men received any colorectal investigation during follow-up, compared with 307,567 (41.4%; 95% CI 41.3% to 41.5%) of men without HIV (adjusted rate ratio 1.15, 95% confidence interval 1.07 to 1.24). When examined according to individual investigation, men with HIV had lower rates of fecal occult blood testing (54.3 versus 71.2 per 1000 person years; adjusted rate ratio 0.74, 95% confidence interval 0.63 to 0.87) and barium enema radiography (2.1 versus. 2.6 per 1000 person years; adjusted rate ratio 0.66, 95% confidence interval 0.39 to 1.12) relative to men without HIV (Figure 1). In contrast, compared with men without HIV, men with HIV had higher rates of flexible sigmoidoscopy (7.0 versus 3.4 per 1000 person-years; adjusted rate ratio 1.72, 95% confidence interval 1.28 to 2.30), rigid sigmoidoscopy (7.6 versus 2.0 per 1000 person-years; adjusted rate ratio 2.98, 95% confidence interval 2.26 to 3.93) and colonoscopy (65.2 versus 47.3 per 1000 person-years; adjusted rate ratio 1.24, 95% confidence interval 1.13 to 1.37) during the follow-up period (Figure 1). These estimates were similar when including those men who had received a colorectal investigation in the preceding five years (Additional file 1: Table S3).

In our secondary analyses examining receipt of colorectal cancer investigations in men with HIV only, 156 (10.9%) and 435 (30.4%) men with HIV underwent either fecal occult blood testing or colonoscopy, respectively. Apart from a higher rate of colonoscopy use among men with a relatively low comorbidity burden (adjusted rate ratio 1.27, 95% confidence interval 1.01 to 1.61), there were no statistically significant associations between the receipt of either of these investigations and patient characteristics (Table 2).

**Table 2:** **Multivariable analysis of predictors of fecal occult blood testing and colonoscopy in men with HIV**

	Outcome; adjusted relative rate* (95% CI)			
**Variable**	**Fecal Occult Blood Tests**		**Colonoscopy**	
Age	1.03	0.99 to 1.07	1.02	0.99 to 1.04
Income Quintile				
1 (reference)	1.00		1.00	
2	1.09	0.71 to 1.67	0.92	0.70 to 1.21
3	0.95	0.58 to 1.55	1.03	0.77 to 1.37
4	1.18	0.73 to 1.91	0.83	0.60 to 1.15
5	0.72	0.42 to 1.22	1.13	0.86 to 1.48
Aggregated Diagnosis Groups^†^				
High (>6)	1.00		1.00	
Low	0.87	0.58 to 1.30	1.27	1.01 to 1.61
Resource Utilization Bands^*^				
High (4 or 5)	1.00		1.00	
Low	0.71	0.47 to 1.06	0.96	0.76 to 1.22
Geographic Residence				
Urban	1.00		1.00	
Rural	0.88	0.41 to 1.89	0.75	0.45 to 1.24
Gastroenterologist visit				
None	1.00		1.00	
>1	0.63	0.26 to 1.53	1.25	0.85 to 1.84
Number of physician visits	1.00	0.99 to 1.01	1.00	0.99 to 1.00

^**†**^Aggregated Diagnosis Groups: Measures levels of patient co-morbidity, with increasing number representing higher level of comorbidity burden.

^*^Resource Utilization Bands: Measures category of expected resource use, from 0 (lowest expected resource use) to 5 (highest expected resource use).

## Discussion

In our population-based study, we found that the majority of men who were eligible for colorectal cancer screening received no such testing during five years of follow-up Although our estimates encompass both screening and diagnostic testing, the receipt of a colorectal investigation by only 49% of men with HIV is clearly sub-optimal. This finding indicates that half of the men with HIV underwent no evaluation of any type and thus were not screened for colorectal cancer. Our findings are similar to those of other studies examining colorectal cancer screening among persons with HIV. In single-centre studies, the proportions of persons with HIV aged 50 years and older who received colorectal cancer screening has ranged from 46.9% to 55.6% [14-17]. In a large study using United States Medicaid claims data on beneficiaries in five states, only 35.8% of 22,928 age-eligible persons with HIV underwent colorectal cancer screening between 1999 and 2007 [18]. However, these findings likely underestimate the extent of screening somewhat because claims for fecal occult blood testing were not examined in this study [18]. Nonetheless, taken as a whole, the existing evidence suggests that efforts to improve colorectal cancer screening rates in persons with HIV are needed.

Our findings have important implications for the care of patients with HIV. Although overall rates of screening were slightly higher in men with HIV relative to men without HIV, small studies suggest that colorectal cancer is diagnosed at a later stage and is associated with poorer outcomes in patients with HIV relative to non-HIV-infected patients [6-10]. In addition, several studies have noted a higher prevalence of adenomatous polyps in HIV-infected patients relative to HIV-negative individuals or population estimates of this outcome [30-33], although such differences have not been observed in all studies [34]. However, authors of these reports universally endorse screening for colorectal cancer in persons with HIV as mechanism for early detection and management of colonic neoplasms [30-34]. We also observed higher rates of colonoscopy utilization among men with HIV with the lowest level of comorbidity. This disparity may have important clinical implications for patients with HIV, since one study noted that 15.7% of all neoplastic lesions and 88.9% of advanced neoplastic lesions in the proximal colon of HIV-infected subjects would have been missed if flexible sigmoidoscopy had been used instead of colonoscopy [30]. It is therefore possible that colonoscopy may be the most appropriate screening method for these patients, although further research is required to delineate best colorectal cancer screening practices in this population.

Although we cannot ascertain the reasons for underutilization of screening in our study, several studies have identified barriers to the uptake of colorectal cancer screening in the general population. Among patients, test-specific barriers related to handling stool and apprehension about bowel preparation, are commonly cited reasons for avoiding colorectal cancer screening [35-37]. In addition, failure of a clinician to suggest screening and lack of knowledge regarding the need for testing were ranked as the two most important barriers to screening in a survey of 3,357 patients [36]. Other reasons cited by individuals for not being screened include fear of a positive test result, lack of confidence in the ability to properly collect specimens for fecal occult blood testing, inadequate explanation by their provider and competing health priorities [38-42]. Furthermore, less education, language barriers and cultural beliefs and attitudes towards screening have been identified as reasons for lower participation in colorectal cancer screening programs among some ethnic groups [43,44]. An earlier study conducted in Ontario using the same datasets as this study noted a significant socioeconomic gradient in colorectal cancer screening, with individuals in the highest income quintile neighborhoods having higher odds of receiving any colorectal cancer investigation [25]. Barriers impeding physician recommendation of colorectal cancer screening to patients have also been identified, and include the limited period of time to address multiple patient comorbidities within a single appointment, forgetfulness, lack of reminders within the health care system and language barriers with non-English speaking patients [45]. In addition, Ontario physicians appear to favour colonoscopy over fecal occult blood testing for colorectal cancer screening, which may influence the nature of testing recommended [46]. Factors associated with colorectal cancer screening among persons with HIV have been investigated in two small studies. In one study of 115 HIV-positive patients, having a primary care physician was associated with an over four-fold increase in the odds of undergoing colorectal cancer screening (odds ratio 4.59, 95% confidence interval 2.01 to 10.48) [16]. In another study, the most commonly cited barriers to colorectal cancer screening among 51 patients with HIV were the precedence of competing health concerns, the time required for testing and fear of the procedure or preparation [47].

Apart from low overall rates of screening among this cohort of men, we found that men with HIV had higher rates of colonoscopy and sigmoidoscopy and lower rates of barium enema radiography and fecal occult blood testing relative to non-HIV-infected men. These discrepancies were not explained by differences in comorbidity, health service utilization, region of residence or neighborhood income quintile. We speculate that the higher utilization of endoscopic investigations among men with HIV reflects a higher likelihood of gastrointestinal symptomatology and diagnostic investigations among these men relative to the non-HIV-infected group, a premise which is supported by previous research. Specifically, in one retrospective clinic-based study examining colorectal cancer screening in patients with HIV, 97% of whom were men, 3.8% of colonoscopies and 64.3% of flexible sigmoidoscopies were undertaken for routine screening, with the remainder being performed for a clinical indication [14]. The spectrum of clinical illness which may predispose patients with HIV to greater utilization of endoscopic testing is broad, and includes chronic diarrhea and non-specific symptoms (e.g. rectal bleeding) associated with human papilloma virus-related anal dysplasia [48,49], both of which occur with greater frequency in persons with HIV relative to the general population [50,51]. In addition, men with HIV in our cohort were more likely to have been seen by a gastroenterologist than HIV-negative men, which may also contribute to the greater use of endoscopic investigations among these patients. Finally, as noted earlier, physicians in Ontario generally favour colonoscopy over fecal occult blood testing for colorectal cancer screening [46]. Whether this influences physician preference when decisions are made about screening for patients with HIV is unknown and is a subject for future research.

The main strength of our study is the use of comprehensive administrative databases, thereby providing population-based estimates of colorectal cancer screening that pertain to all men aged 50 to 65 years who have been diagnosed with HIV. To our knowledge, ours is the first population-based study examining rates of colorectal cancer screening in these men and addresses some limitations of earlier studies addressing this question, including lack of generalizability. However, several limitations of our work merit emphasis. First, our findings are not generalizable to the population of women with HIV and men below the age of 50. This limitation is shared by previous research examining colorectal cancer screening [14]. In addition, despite excluding men with a history of colorectal cancer, inflammatory bowel disease and colorectal investigations in the five years preceding cohort entry, we could not discriminate between screening and diagnostic testing using our databases. However, because our estimate includes all testing performed, our finding that 49% of men with HIV received any colorectal cancer testing is an upper bound on the proportion of men screened, reinforcing the underutilization of colorectal cancer screening in these patients.

## Conclusions

In conclusion, we found that despite universal access to colorectal cancer screening, receipt of this preventive modality among men over the age of 50 is suboptimal. Although overall rates of screening were slightly higher among men with HIV, interventions are required to further optimize uptake of colorectal cancer screening in these patients. Further research is necessary to understand reasons for the suboptimal uptake of colorectal cancer screening in persons with HIV determine the incidence of colorectal cancer in this population and ascertain whether existing screening guidelines are applicable in the setting of HIV infection.

## Additional file

Additional file 1: Table S1. Procedural and diagnostic codes for colorectal investigations. **Table S2**. Diagnostic codes for colorectal cancer and inflammatory bowel disease. **Table S3**. Adjusted rate ratios for receipt of colorectal investigations in men with HIV relative to non-HIV-infected men (including men who had previously received a colorectal investigation in the 5 years preceding the index date).

## Abbreviations

HIV: Human immunodeficiency virus
ICD-9: International classifications of diseases 9th edition
ADGs: Aggregated diagnosis groups
RUBs: Resource utilization bands
