# Supplementary material for: A population-based study of the extent of colorectal cancer screening in men with HIV
Source: BMC Health Serv Res. 2015 Feb 1;15:51. doi: 10.1186/s12913-015-0711-9 (PMC4318126; doi:10.1186/s12913-015-0711-9)
Supplement: Additional file 1: Table S1. — Procedural and diagnostic codes for colorectal investigations. Table S2. Diagnostic codes for colorectal cancer and inflammatory bowel disease. Table S3. Adjusted rate ratios for receipt of colorectal investigations in men with HIV relative to non-HIV-infected men (including men who had previously received a colorectal investigation in the 5 years preceding the index date). [file 12913_2015_711_MOESM1_ESM.doc]

**Additional file 1**

**Table S1: Procedural and diagnostic codes for colorectal investigations**

|  | Ontario Health Insurance Plan (OHIP) Databse | Canadian Institute for Health Information Discharge Abstract Database (CIHI DAD) |
| --- | --- | --- |
| Fecal occult blood testing | L181, G004 |  |
| Rigid sigmoidoscopy | Z535, Z536 | 01.24 |
| Flexible sigmoidoscopy | Z580, Z555 (without E740, E741, E747 or E705 on same day) |  |
| Barium enema radiography | X112, X113 |  |
| Colonoscopy | Z555 + one of E740 or E741 or E747 or E705 on same day | 01.2, 01.22 |

**Table S2: Diagnostic codes for colorectal cancer and inflammatory bowel disease**

|  | Ontario Cancer Registry (OCR) | Ontario Health Insurance Plan (OHIP) Database | Canadian Institute for Health Information Discharge Abstract Database (CIHI DAD) |
| --- | --- | --- | --- |
| Colorectal and anal cancer | 153.x, 154.x |  |  |
| Inflammatory bowel disease (Crohn’s disease and ulcerative colitis) |  | 555.0 to 555.9, 556.0 to 556.9 | K500, K501, K508 to K515 |

**Table S3: Adjusted rate ratios for receipt of colorectal investigations in men with HIV relative to non-HIV-infected men (including men who had previously received a colorectal investigation in the 5 years preceding the index date)**

| **Colorectal Investigation Type** | **Adjusted relative rate** | **95% confidence interval** |
| --- | --- | --- |
| Fecal occult blood testing | 0.68 | 0.60 to 0.76 |
| Flexible sigmoidoscopy | 1.47 | 1.20 to 1.82 |
| Rigid sigmoidoscopy | 3.68 | 3.13 to 4.32 |
| Colonoscopy | 1.23 | 1.15 to 1.31 |
